# The Impact of Demographics, Life and Work Circumstances on College and University Instructors’ Well-Being During Quaranteaching

**DOI:** 10.3389/fpsyg.2021.643229

**Published:** 2021-06-11

**Authors:** Magdalena Jelińska, Michał B. Paradowski

**Affiliations:** ^1^Institute of Applied Linguistics, University of Warsaw, Warsaw, Poland; ^2^Institute of Linguistics, University of Silesia, Sosnowiec, Poland

**Keywords:** teacher well-being, emergency remote instruction, COVID-19 pandemic, negative affect, situational anxiety, loneliness, higher education, work-life synergy

## Abstract

In response to the outbreak of the COVID-19 pandemic, educational institutions around the world were forced into lockdown in order to contain the spread of the virus. To ensure continuous provision of education, most transitioned to emergency remote instruction. This has been particularly the case in higher education (HE) institutions. The circumstances of the pandemic have brought unprecedented psychological pressure on the population, in the case of educators and students exacerbated by the transition to a mode of instruction that was completely novel to the majority. The present study examines how college and university instructors dealt with teaching online in these unparalleled circumstances, with a focus on how factors connected with their daily lives and livelihoods influenced their well-being. Between April and September 2020, a comprehensive online survey was filled out by 804 HE instructors from 92 countries. We explore how sociodemographic variables such as gender, age, relationship status, living conditions, and length of professional experience non-trivially affect situational anxiety, work-life synergy, coping, and productivity. The results contribute to a better understanding of the impact of the pandemic and emergency remote instruction on college and university instructors’ well-being by explaining the mechanisms mediating the relationship between individual, contextual, and affective variables. It may provide helpful guidelines for college and university administrators as well as teachers themselves as to how help alleviate the adverse effects of the continuing pandemic and possible similar disruptions leading to school closures on coping and well-being.

## Introduction

The circumstances of the pandemic have brought unprecedented psychological pressure on the population. The adverse—sometimes long-lasting—psychological impact of the lockdown restrictions, stay-at-home orders, quarantine, and other repercussions span anxiety, post-traumatic stress symptoms, psychological distress, confusion, panic disorder, anger, depression, insomnia, and emotional exhaustion (Brooks et al., 2020; Qiu et al., 2020; Cénat et al., 2021a).

Teachers and students have been among the more impacted groups (International Labour Organization, 2020). As campuses almost all over the world successively shut down, in order to ensure the continuity of learning and of communication between teachers and students (Karalis, 2020), educators were thrust into the provision of alternative modes of delivery, or “emergency remote teaching” (Hodges et al., 2020; Reimers and Schleicher, 2020) that for most was an entirely novel form of work. Often, the anxieties were exacerbated by job uncertainty, especially for faculty on precarious contracts who—as happened for instance across the United Kingdom—were often dismissed by their institutions in an attempt to cut costs (World Bank, 2020:10).

The combined effect of lockdowns and the transition to online delivery have severely affected teacher and student coping and well-being. The strong possibility of deteriorating mental health because of the sense of uncertainty and anxiety among students and faculty members was understandably envisaged already in the initial stages of the pandemic (Sahu, 2020).

## Literature Review

The impact of the COVID-19 pandemic on higher education has been a burgeoning topic of discussion (Fischer, 2020). However, the majority of the extant literature concerned with “pandemic pedagogy” tends to focus on the logistics and provision of training (Reimers and Schleicher, 2020), without taking into account the psychological impact of the situation on the teachers’ functioning, let alone trying to tease apart the relative influence of the factors affecting instructors’ well-being. For instance, the University of Houston (2020) published a report summarizing the faculty’s perceptions regarding the transition to a remote teaching model, revealing significant variation in terms of the implementation of technology tools and of the mode of instruction. Watermeyer et al. (2021) carried out a survey of academic teachers’ reaction to the move to online teaching, and found that the majority of the respondents felt confident or strongly confident in their ability to facilitate online teaching and assessment and considered their institutions to be supportive in facilitating the move to online delivery. Bensaid and Brahimi (2021) likewise attributed the successful maintenance of the learning cycle in higher education institutions in the Gulf to their already established distance education, swift administration and policy steps, and access to resources. Jelińska and Paradowski (2021b) in turn revealed how teachers’ perception of how their students were coping with the novel situation was influenced by their own demographics and professional adaptation to emergency remote teaching.

Where the psychological impact of the pandemic in education was a concern, the existing literature has tended to focus on the student population. A topical analysis of tweets (Duong et al., 2020) revealed that negative sentiments toward the central issues of COVID-19 were significantly higher among students than in the general population. Khodabakhshi-Koolaee (2020) in interviews with 15 postgrad students in Iran who had experienced living in quarantine, as three of the four main emerging themes identified (i) developing negative emotions, confusion and pessimism, (ii) concerns about family health, as well as (iii) economic and social concerns and “fear of tomorrow.” In a qualitative phenomenological study with five university students in the Philippines, Alvarez (2020) showed how learning engagement and isolation and the lack of affective and emotional support affected learning engagement. Another study (Yang et al., 2021) showed that students’ self-efficacy and well-being were positively predicted by their perceived closeness with the teacher, peer influence, and perceived control over own learning. Students’ perceived learning outcomes were also found to be positively influenced by instructional support and instructor innovation, and–interestingly–negatively by teacher performance (Wang et al., 2021). In a multi-national survey of undergraduate students, Nguyen et al. (2021) reported that the majority preferred synchronous classes, which mode–especially when it involved active-learning techniques–correlated with higher levels of engagement and motivation. Student engagement and adaptability were also found to significantly correlate with academic emotion (Zhang et al., 2021). An analysis carried out at a university in Russia (Dikaya et al., 2021) revealed an association between students’ attitudes to forced remote learning and their interpersonal communicative skills and thinking and learning styles. A survey of students’ emotions during and perceptions of the shift to online teaching at a Greek university (Karalis and Raikou, 2020) found that although the majority of the students were satisfied with the new way of attending classes, upon the closure of the university over three quarters had felt negative emotions such as stress and anxiety about how the studies would be completed, fear of the possibility of non-continuation of studies, and/or sadness about the interruption; after the online classes had started, some of the initial negative emotions gave way to an increase in relief that the semester would not be lost, and joy at the continuity of the classes. Across the Turkish border, students reported high perceived stress, mild generalized anxiety, and low satisfaction with life (Aslan et al., 2020). In a questionnaire probing the mental health of medical college students, Cao et al. (2020) found that risk factors increasing anxiety were economic consequences as well as having relatives or acquaintances infected with SARS-CoV-2, while social support, living with parents, and family income stability were protective factors. Similar results were obtained by Wilczewski et al. (2021), whose investigation of the psychological and academic effects of learning online among international students enrolled at the University of Warsaw revealed that those who had returned to their home countries (and therefore likely had familial support) exhibited higher academic adjustment, while quarantine and self-isolation increased the levels of loneliness – and acculturative stress when in the host country. Amendola et al. (2021) study carried out among students in Switzerland showed that anxiety symptoms decreased with time, and its levels were predicted by older age, female gender, out-of-country nationality, loneliness, concerns about own health (positively), and resilience and social support (negatively). Awoke et al. (2021) measured perceived stress among health science students in Ethiopia, with identified correlates of the construct including rare contacts with friends and a decreased household income. In a study employing interview surveys with 195 university students in the United States, over seventy per cent of the participants indicated increased stress and anxiety due to the COVID-19 outbreak. The identified factors that contributed to the increased levels of stress, anxiety, and depressive thoughts among the students included among others fear and worry about their own health and the health of their loved ones, difficulty in concentrating, disruptions to sleeping patterns, decreased social interactions due to physical distancing, and increased concerns regarding academic performance. To cope with stress and anxiety, participants sought support from others and helped themselves by adopting either negative or positive coping mechanisms. Clabaugh et al. (2021) found that levels of stress and difficulty with coping with pandemic disruptions were related to neuroticism, an external locus of control, gender, and ethnicity, while a study from Mexico (Gaeta et al., 2021) discovered a relationship between university students’ self-regulated learning and emotions such as tranquillity, hope, gratitude, joy, loneliness, and disinterest, mediated by coping strategies. A study by Alemany-Arrebola et al. (2020) showed that college students with a higher level of anxiety expressed more negative emotions and declared lower academic self-efficacy. A World Bank report on tertiary education during the pandemic recognized that “[l]ong periods of self-isolation can have an adverse impact on the psychological well-being of students and staff, especially for those who live alone, international students, and students/staff who are not in their place of origin” (2020:6).

On the teacher front, Jelińska and Paradowski (2021a) demonstrated how educators’ engagement in and coping with remote instruction were moderated by gender, teaching level, mode of delivery (synchronous vs. asynchronous), and the economic status of the respective countries, while MacIntyre et al. (2020) investigated the correlates of approach and avoidant coping strategies among an international sample of language teachers during the conversion to online instruction. Our own analysis of responses from 1,149 language instructors revealed that on average, they found that the remote mode of delivery depressed students’ language progress by around 64% (!), with concern about students’ outcomes most prominent in beginner-level classes (Paradowski and Jeliǹska, under review a). Elsewhere (Paradowski and Jelińska, under review b) we analyzed how inequalities among educators related to demographics, family support, access to resources and infrastructure, and anxieties about the future influenced their psychological overload, and how this influence was mediated by their perception of student coping. We also identified predictors of stress among 435 linguistics instructors teaching online, revealing the influence of anxiety about the future, living conditions, self-acceptance, appraisal of the situational impact, course optionality, and perceived effectiveness of the virtual mode of delivery, with a mediating effect of acceptance of the virtual instructional mode (Paradowski and Jelińska, under review c). A study carried out after school reopening among teachers from the Basque Country (Ozamiz-Etxebarria et al., 2021) showed that levels of anxiety, depression and stress symptoms were influenced by gender, age, job stability, the level of education taught, and parental status. In the Philippines, Rabacal et al. (2020) revealed a moderate impact of COVID-19 on teachers’ quality of life, while Oducado et al. (2020) found that more than half of the teachers surveyed experienced moderate stress related to the epidemic, but these authors focused primarily on the health factor. Otherwise, however, studies examining the relationships between instructors’ adaptation to and well-being during the crisis teaching period and background variables have been scarce. This contribution intends to help fill the lacuna by investigating how university instructors’ sociodemographic characteristics as well as life and work circumstances affected their coping and wellness.

Wellbeing constitutes a complex and multi-dimensional construct related to life satisfaction, resilience, work outcomes, more adequate regulation strategies and better health (Keyes, 1998; Diener et al., 1999; Diener, 2009; Dodge et al., 2012; Fisher, 2014), and is defined in various ways generally within either a *hedonic* or a *eudaimonic* theoretical approach (Kesebir and Diener, 2008). According to the *hedonic* tradition, subjective wellbeing constitutes “a person’s cognitive and affective evaluations of his or her life” (Diener et al., 2003:63). The cognitive component reflects a sense of satisfaction with life, whereas the emotional element is composed of high positive and low negative affect referring to moods and emotions (e.g., Bradburn, 1969; Diener, 1984, 2006; Diener and Suh, 1997:200; Diener et al., 1999; Kahneman et al., 1999; Lyubomirsky and Lepper, 1999; Arthaud-Day et al., 2005; Kim-Prieto et al., 2005; De Leersnyder et al., 2013). The *eudaimonic* tradition in turn relies on a notion of psychological wellbeing which refers to positive psychological functioning and development covering purpose in life, autonomy, personal growth, self-esteem, acceptance, mastery, control, and positive relations with others (e.g., Rogers, 1961; Ryff, 1989a, b; Waterman, 1993; Keyes, 1998; Ryan and Deci, 2001; Keyes et al., 2002; Ryff and Singer, 2008). This theoretical approach also includes emotional and affect regulation (Korpela et al., 2018; Puente-Martínez et al., 2018), which are linked to positive self-image (including enhanced self-esteem), situation management, and relatedness (Koole, 2009).

In most theories, a key indicator of wellbeing is *positive* affect (Lyubomirsky et al., 2005; Cohn and Fredrickson, 2009; Kong and Zhao, 2013; Coffey et al., 2014; Szczygieł and Mikolajczak, 2017). However, existing *negative* emotions and mood should not be ignored as emotional wellbeing has been defined as “the ratio of positive affect (PA) to negative affect (NA) in a person’s life over a representative time period” (Larsen, 2009:249). Studies indicate that the amounts of positive and negative affect are uncorrelated (e.g., Diener and Emmons, 1985; Schmukle et al., 2002), and that their relative contribution to emotional wellbeing varies (e.g., Larsen et al., 1990).

Many researchers have indicated that the negative affect system is more reactive than the positive affect system (e.g., Ito et al., 1998; Cacioppo and Gardner, 1999; Ito and Cacioppo, 2005; Grinde, 2012). Moreover, Larsen (2002) indicated that the same levels of objectively bad and good events cause respectively higher levels of negative than positive affect. As observed by Musch and Klauer (2003), compared with positive ones, negative events engage more attentional resources and are stored more accessibly in memory. These findings lead to the conclusion that negative affect is stronger than positive affect with respect to its reactivity, duration, and cognitive processing (Larsen, 2002, 2009; Larsen and Prizmic, 2008). Individuals tend to pay more attention to negative information compared to positive information, processing and recalling the former more thoroughly (Baumeister et al., 2001; Rozin and Royzman, 2001). As a result, negative emotions and mood influence overall emotional wellbeing to a greater extent than positive ones (Larsen, 2009). For this reason, Grinde (2016) suggested that negative feelings should be included in wellbeing measures. In this study, we focus on this aspect of emotional wellbeing.

In the ongoing discussion about the relevant definition of well-being, Dodge et al. (2012:230) took into account various existing conceptualizations and characterized wellbeing as the balance between individual psychological, social and/or physical resources and psychological, social and/or physical challenges encountered in everyday life. The challenges introduce an imbalance, which in turn reduces wellbeing. To restore the equilibrium and regain their wellbeing, an individual needs to use or adapt all their resources.

This approach seems to be particularly relevant in the context of the COVID-19 pandemic and crisis shifts in life such as the abrupt transition to remote teaching, which are potential challenges disturbing teachers’ wellbeing. Studies on past infectious disease outbreaks have shown that such health emergencies impact survivors’, their families’, and affected communities’ mental health and can lead to higher levels of anxiety, PTSD, insomnia, and depression (Mohammed et al., 2015; Keita et al., 2017). Confinement and social and physical distancing can additionally exacerbate the negative symptoms and require additional relevant individual resources in order to re-establish the balance and protect wellbeing. A body of research from the COVID-19 pandemic has accordingly identified numerous stressors and risk factors for mental health, spanning but not limited to fear of or actually getting infected (Bo et al., 2020; Nguyen et al., 2020; Rogers et al., 2020), inadequate information as well as excessive consumption of negative information from social media (Gao et al., 2020), the experience of quarantine (Lei et al., 2020), infection and/or death of loved ones, stigma, social isolation and loneliness, frustration, boredom, job/wage losses and associated financial insecurities (Brooks et al., 2020; Cénat et al., 2020, 2021b; Nicola et al., 2020), among others. Not surprisingly, studies have reported increased levels of anxiety (Lee et al., 2020; Lei et al., 2020; Moghanibashi-Mansourieh, 2020), distress (Hao et al., 2020; Mazza et al., 2020), post-traumatic stress (Bo et al., 2020; Liu et al., 2020), depression (Lei et al., 2020; Nguyen et al., 2020), insomnia (Li et al., 2020), and other dimensions of psychological impact (Ahmed et al., 2020; Cao et al., 2020; Moccia et al., 2020; Xiong et al., 2020; Cénat et al., 2021a, b; Huang and Zhao, 2021) in pandemic-affected populations.

Teachers’ work is considered to be one of the most stressful professions (Frenzel et al., 2016; MacIntyre et al., 2019). Many researchers have observed that teaching is related to a lower-than-average level of mental health, poorer physical health, and lower job satisfaction, making teachers particularly vulnerable to burnout (Johnson et al., 2005; Chang, 2009; Brackett et al., 2010; Keller et al., 2014; MacIntyre et al., 2019). All these factors may significantly reduce teachers’ subjective wellbeing (Kieschke and Schaarschmidt, 2008; Woolfolk Hoy, 2008) and decrease their work effectiveness and learners’ outcomes (Klusmann et al., 2008; Day and Gu, 2009; Frenzel et al., 2016; Lee et al., 2016). In addition, in the context of the COVID-19 pandemic the conditions of living and working under lockdown could induce negative feelings and increase teachers’ negative affect. An online poll of 1,122 US faculty members conducted in October 2020 (Business Wire, 2021) revealed that more than twice as many respondents were feeling stressed, fatigued, and angry compared with the year before.

The COVID-19 pandemic enforced various challenges such as remote work, limited social interactions, and social isolation, provoking feelings of situational loneliness, which may negatively affect health and wellbeing (DiGiovanni et al., 2004; Cacioppo et al., 2010; Lin et al., 2010; Theeke, 2010; Beutel et al., 2017; Mullen et al., 2019; Son et al., 2020; Groarke et al., 2020; Nguyen et al., 2021). In the United Kingdom, 36% of adult respondents declared feeling lonely sometimes or often during the epidemic (Li and Wang, 2020). Higher loneliness was related to physical distancing and reduced social contact (Losada-Baltar et al., 2021). It was also associated with financial concerns and worries about the impact of prolonged quarantine as well as feelings of fear and uncertainty, increased depression, anxiety, stress, and an increased affective response to different aspect of the pandemic in Poland, the United Kingdom, and the United States (Brooks et al., 2020; Holmes et al., 2020; Jia et al., 2020; Kantor and Kantor, 2020; Killgore et al., 2020; Okruszek et al., 2020; Smith and Lim, 2020; Son et al., 2020; Cacioppo et al., 2021). Studies indicate that more frequent face-to-face contacts (unlike remote or virtual interactions), as well as closeness and quality of relationships moderate the negative loneliness-inducing influence of the COVID-19 pandemic and function as protective factors (Bu et al., 2020a, b; Groarke et al., 2020; Li and Wang, 2020; Tull et al., 2020; Rosenberg et al., 2021). Close, frequent and satisfying relationships constitute a basic human need and are core indicators of the social aspect of wellbeing (Baumeister and Leary, 1995; Ryan and Deci, 2000; Seligman, 2011).

The aforementioned results concern the general population. However, there is little evidence how situational anxiety and loneliness and anxiety as well as closeness of social relationships, which turned out to be significant determinants of functioning during the COVID-19 pandemic, influence the wellbeing of teachers, especially in the context of a challenge (Dodge et al., 2012) such as an emergency transition to distance instruction. In this study we examine the importance of these variables in the emotional aspect of wellbeing, alongside other factors such as perceived situational coping, work-life synergy (as an indicator of work/life satisfaction protecting against burnout) and self-perceived productivity (efficacy). We also take into account other individual factors and capital, such as teachers’ age, gender, relationship status, and years of experience, which may play a particular role in protecting wellbeing (*op. cit.*).

The aim of this study is accordingly to investigate factors influencing higher education (HE) teachers’ negative affect as a substantial component of emotional wellbeing (Larsen, 2002, 2009; also: Ito et al., 1998; Cacioppo and Gardner, 1999; Ito and Cacioppo, 2005; Larsen and Prizmic, 2008; Grinde, 2012) during the first few months of the COVID-19 pandemic in the context of adaptation to emergency remote teaching, thus potentially conditions more stressful than during “business as usual.” Based on Dodge et al.’s (2012) resources–challenges balance approach to wellbeing and factoring in current research pointing to situational loneliness, anxiety and social relationships as among the most significant factors influencing mental health and wellbeing during the COVID-19 pandemic, we examine their role in the professional group of HE instructors during a time of heightened vulnerability to stress and its various consequences. In line with the recommendations by Bricheno et al. (2009), apart from subjective indicators we also include objective factors potentially determining wellbeing such as gender, age, relationship status, living conditions, and the number of years of teaching experience.

The study is guided by the following exploratory research questions:

**RQ1:** To what extent did HE teachers’ negative affect vary depending on sociodemographic factors such as: (1a) gender, (1b) age, (1c) relationship status, (1d) living conditions, and (1e) length of experience in teaching?

**RQ2:** Which of the factors potentially affecting professional adaptation to emergency remote instruction in the COVID-19-related context, from among: (2a) situational loneliness, (2b) situational anxiety, (2c) family and social support, (2d) perceived self-productivity, (2e) situational coping, (2f) work-life synergy, and (2g) sociodemographic variables such as gender, age, relationship status, living conditions and length of experience in teaching, are associated with teachers’ negative affect, and to what extent?

**RQ3:** What is the relative contribution of the respective predictors and to what extent does each of them determine teachers’ negative affect and, consequently, emotional wellbeing?

## Materials and Methods

### Participants

In this contribution, we focus on survey respondents from higher education institutions. From April through September 2020 a total of 804^1^ HE instructors participated in the study. More than 80% were teaching at a university or graduate school. The instructors hailed from 6 continents and 92 countries and autonomous territories, half of them (52%) from Europe (Table 1). The mean reported age was 44.1 years (*SD* = 12.5), with over half the respondents aged between 36 and 55. Most did not report the length of their professional experience, 23% had been teaching for more than 5 years (*M* = 2.5, *SD* = 1.60), whereas 18.5% less than that. During the COVID-19 pandemic approximately three quarters were teaching synchronous classes in real time, despite more than two thirds reporting no prior experience with remote instruction.

**TABLE 1:** Socio-demographic characteristics of the participants (*N* = 804).

	Frequency (n)	Percent (%)
**School type**		
community college/college/undergraduate school	109	13.5
university/graduate school	651	81.0
teacher training college	17	2.1
foreign language center affiliated with a university	27	3.4
**Continent**		
**Europe**	419	52.1
(Austria, Belgium, Belarus, Bulgaria, Czech Republic, Croatia, Cyprus, Denmark, Finland, France, Georgia, Germany, Greece, Hungary, Ireland, Italy, Jersey, Lithuania, Luxembourg, Malta, Montenegro, Netherlands, Norway, Poland, Portugal, Romania, Russia, Serbia, Slovakia, Spain, Sweden, Switzerland, United Kingdom, Ukraine)		
**Asia**	122	15.2
(Bahrain, Bangladesh, Brunei, Cambodia, China, Hong Kong, Macao, India, Indonesia, Iraq, Israel, Japan, Jordan, Kuwait, Laos, Lebanon, Malaysia, Nepal, Oman, Pakistan, Palestine, Philippines, Qatar, Saudi Arabia, South Korea, Sri Lanka, Taiwan, Thailand, Turkey, United Arab Emirates, Vietnam)		
**North America**	210	26.1
(Bahamas, Canada, Costa Rica, Mexico, Puerto Rico, United States)		
**Oceania**	25	3.1
(Australia, Fiji, Kiribati, Aotearoa/New Zealand)		
**Africa**	17	2.1
(Algeria, Egypt, Kenya, Lesotho, Malawi, Mauritius, Morocco, Senegal, South Africa, Tunisia)		
**South America**	11	1.4
(Argentina, Brazil, Chile, Colombia, Peru, Trinidad and Tobago, Uruguay)		
**Age group (years)**		
<25	39	4.9
25 – 35	163	20.3
36 – 45	229	28.5
46 – 55	219	27.2
56 – 65	128	15.9
>65	25	3.1
not reported	1	0.1
**Years of experience**		
<5 years	149	18.5
6 –10 years	49	6.1
11 – 15 years	42	5.2
16 – 20 years	24	3.0
>20 years	70	8.7
not reported	470	58.5
**Gender^2^**		
female	578	71.89
male	215	26.74
non-binary/not listed	11	1.37
**Relationship status**		
in a relationship	579	72.01
single	207	25.75
not reported	18	2.24
**Prior experience with remote teaching**		
lack of experience	541	67.29
prior experience	265	32.71
**COVID-19 cases among close others**		
no	693	86.19
yes	111	13.81
**COVID-19-related deaths among close others**		
no	776	96.52
yes	28	3.48

*^2^Participants who selected the “Non-binary/Not listed” option as their gender constituted only 1.4% of the respondents and thus their gender was not included as a variable in the inferential analyses.*

Almost 72% of the participants were female. A similar percent declared to live with their families or partners. Approximately 14% had a close other who had contracted COVID-19, and 3.5% experienced the death of a close person due to the virus.

### Measures

To evaluate how the epidemic reality and remote teaching conditions affect instructors’ wellbeing, we devised an online survey composed of 441 items (Supplementary Material). It included items concerning respondents’ sociodemographics, the circumstances surrounding the participants’ transition to emergency remote instruction, their personal experiences, behaviors, attitudes, feelings, physical and mental health, and personality traits. To measure psychological constructs, 23 short scales were developed from International Personality Item Pool [IPIP] (2018), International Personality Item Pool (n.d.) items. In order to glean more multidimensional information related to the specific circumstances of the situation that could not be properly measured with existing batteries, we complemented these with custom-made scales, single-item indicators, as well as open-ended questions.

In the following analysis we focus on the relative contribution of demographic variables as well as factors measured with four short scales (Supplementary Appendix 2) and three single-item indicators:

***Negative affect*** was measured with 11 items assessing to what extent instructors felt negative emotional states such as sadness (e.g., “I have been sad”), irritation (e.g., “I have been feeling irritable”), strain (e.g., “I feel building up pressure”), and emotional instability (e.g., “I have been having bouts of anxiety/panic attacks”) as well as symptoms of fatigue (e.g., “I feel tired during the day”). Items in this and the other scales were answered on a six-point Likert scale ranging from completely disagree to completely agree. The internal consistency of this scale was high (Cronbach’s α and McDonald’s ω_*h*_ = 0.92, Guttman’s λ_6_ = 0.90). Positive correlations (*r* = 0.75, *p* < 0.05) with perceived stress measured by the Perceived Stress Scale (PSS; Cohen et al., 1983) and negative (*r* = –0.45, *p* < 0.05) with self-compassion measured with the short form of the Self-Compassion Scale (Raes et al., 2011) corroborate good convergent validity of the scale developed for this study.

***Situational loneliness*** was measured with a 3-item scale assessing the extent to which teachers felt lonely (e.g., “I feel lonely”) and lacked contact with their colleagues (e.g., “I miss daily conversation with my colleagues”) during the period of lockdown. In the current study its internal consistency reached Cronbach’s α = 0.86, McDonald’s ω_*h*_ = 0.85, and Guttman’s λ_6_ = 0.84; however, given its short length, further studies may be needed to verify its validity.

***Situational anxiety*** caused by the COVID-19 pandemic was assessed with a 5-item scale measuring to what extent instructors felt worried about different aspects of their living conditions such as their future, job stability, housing conditions, economic situation, and concern about their family members (e.g., “I worry about my job stability”). The scale showed good internal consistency of Cronbach’s α = 0.81, McDonald’s ω_*h*_ = 0.82, and Guttman’s λ_6_ = 0.80. Positive correlation (*r* = 0.47, *p* < 0.05) with perceived stress measured with the Perceived Stress Scale (PSS; Cohen et al., 1983) indicates relatively good convergent validity of the developed situational anxiety scale.

***Family and social support*** was assessed with 4 items measuring to what extent the teachers felt the support and closeness of their families and significant others (e.g., “I have good relations at home,” “I feel comfortable having my family/partner/roommates/flatmates around during this time”). The scale reported good internal consistency of Cronbach’s α and McDonald’s ω_*h*_ = 0.80, and Guttman’s λ_6_ = 0.78.

The single-item indicators concerned situational coping, work-life synergy and self-productivity, complemented with sociodemographic information such as teachers’ age, gender, relationship status, and years of professional experience. We applied the comprehensive single item approach (CSI) proposed by Konstabel et al. (2012, 2017), which assumes that the content validity of a one-item indicator is preserved when this item has a comprehensive content and is newly written instead of being selected from a longer scale. This approach is based on the assumption that every individual has self-knowledge and is able to characterize it if a given construct is sufficiently simple, unambiguous or circumscribed to be comprehensible to the respondent (Wanous and Reichers, 1996; Loo, 2002). This means that the CSI should not be applied to more complex traits or dispositions. Thus, in this research it was only used with generic and more homogenous variables.

### Procedure

The custom-made online questionnaire was active from April until September 2020 on a commercial survey software platform (in order to reach respondents in countries where solutions such as Google Forms cannot be accessed without a VPN). Participant recruitment was carried out with a snowball sampling technique via several channels utilizing the researchers’ direct personal contacts, mailing lists and websites of professional associations, and thematic groups and pages on social media. Participation was voluntary and the respondents were informed about the purpose of the survey. The protocol had received IRB approval.

Eligibility required having transitioned from regular face-to-face classes to online teaching as part of the response to the COVID-19 epidemic. The filter question excluded more than 13% of the initial survey takers – persons who either continued teaching face-to-face, or who had already been teaching online before the school closures happened.

### Data Analysis

To observe the differences between the variables and to answer RQ1a-1e, we calculated a *t*-test for relationship status and one-way ANOVAs for all the remaining sociodemographic variables. To find the answer to RQ2 concerning the extent of the association between such potential factors affecting HE teachers’ professional adaptation to emergency remote instruction in the COVID-19-related context as (2a) situational loneliness, (2b) situational anxiety, (2c) family and social support, (2d) perceived self-productivity, (2e) situational coping, (2f) work-life synergy, and (2g) sociodemographic variables such as: gender, age, relationship status, living conditions, and length of experience in teaching on the one hand, and teachers’ negative affect on the other, we calculated Pearson’s correlation coefficient for continuous variables and Spearman’s ρ for categorical variables. To study the impact of the predictive factors on negative affect as an indicator of teachers’ wellbeing, we used STATISTICA’s General Regression Models (GRM) module for building models containing categorical and continuous predictor variables (analysis of covariance design). As opposed to (multiple) regression models, applicable only to continuous variables, the general linear model permits analyses of any ANCOVA or MANCOVA design which includes both categorical (e.g., gender) and continuous predictor variables as well as a wide variety of different types of design. Analysis of covariance (ANCOVA), defined as a general linear model, combines at least one categorical predictor (i.e., one-way ANOVA) and continuous predictors (i.e., linear regression). The dependent variable(s) in such a general linear model is/are always continuous. Similarly as in the case of regression, using ANCOVA requires meeting five assumptions: normality of residuals, homogeneity of variance, homogeneity of regression slopes, linearity of regression, and independence of error terms (Garson, 2012; Philippas, 2014). In this study, a general linear model was the most suitable statistical tool as it allowed to take into account both categorical and continuous predictors. Applying forward selection, based on adding the most statistically significant variables to the model until there are no variables left meeting the entry criteria and a satisfactory regression equation has been found, underlies the answer to RQ3. This selection method allows building a purely exploratory model, which is not based on any theoretical assumptions, but on the statistically significant impact of the variables.

The linearity assumption was examined via a visual inspection of scatterplots, which indicated that the variables and the residuals of the regression (i.e., the errors between the observed and the predicted values) were normally distributed. The variance inflation factor (VIF) not exceeding 1.6 and tolerance values ranging from .64 to .94 indicated lack of multicollinearity. The lack of collinearity was also confirmed by a matrix of Pearson’s bivariate correlations among all the predictors. A visual analysis of a scatterplot of residuals versus predicted values indicated that the assumption of homoscedasticity was satisfied as well.

The significance level was set at .001 for ANCOVAs and at .05 for the remaining analyses. Effect sizes are reported with Cohen’s *d* for the *t*-test and η_*p*_^2^ for ANOVA, respectively.

## Results

To answer **RQ1** and find out whether and to what extent HE teachers’ negative affect varied depending on sociodemographic factors such as: (1a) gender, (1b) age, (1c) relationship status, (1d) living conditions, and (1e) length of teaching experience, we carried out Student’s *t*-test and ANOVA. Descriptive statistics for teachers’ negative affect with respect to the sociodemographic variables are displayed in Table 2. The results indicate that in the first few months of the COVID-19 pandemic, teachers differed in their wellbeing, which reflected their negative emotional states.

**TABLE 2:** Significant differences in teachers’ negative affect (*N* = 804).

			Negative affect				
		
	M	SE	Effect size	95%CI		*F* or *t*	df
**Gender**			η_*p*_^2^ = **0.01**	**0.002**	**0.03**	**4.67**	**2,801**
female	32.61*	0.53		31.56	33.66		
male	30.53*	0.83		28.89	32.17		
not listed/non-binary	40.73	0.68		32.53	48.92		
**Relationship status**			*d*** = 0.2**	**0.07**	**0.38**	2.46*	**784**
single	34.02*	0.86		32.33	35.72		
in relationship	31.50*	0.53		30.47	32.55		
**Living conditions**			η_*p*_^2^ = **0.01**	**0.001**	**0.02**	4.52*	**2,801**
on my own	34.32*	0.98		32.37	36.26		
with partner	32.30	0.64		31.05	33.56		
with family	30.50*	0.81		28.91	32.09		
**Age group**			η_*p*_^2^ = **0.01**	**0.001**	**0.02**	5.96*	**2,801**
<25	35.33	2.25		30.78	39.89		
25–35	34.07	0.97		32.14	35.99		
36–45^*a*^	34.26	0.86		32.56	35.95		
46–55^*b*^	30.55*	0.85		28.87	32.23		
56–65^*ab*^	28.89*	1.02		26.87	30.92		
>65	26.68	1.84		22.87	30.49		
**Professional experience in years**			η_*p*_^2^ = **0.05**	**0.01**	**0.09**	3.95*	**4,329**
<5 years	33.41	1.11		33.72	38.96		
6 –10 years	34.61	1.49		28.77	33.53		
11 – 15 years	28.10	1.92		27.63	32.22		
16 – 20 years	28.79	2.30		23.17	30.93		
>20 years	28.37	1.23		31.31	33.65		

Superscripts indicate significant pairwise differences based on Tukey’s *post hoc* test (*p* < 0.05) [for age groups: ^*a*^25–35, ^*b*^36–45; for professional experience: ^*c*^<5 years]. *indicates statistical significance at *p* < 0.05; terms and values in bold refer to the influence on teachers’ negative affect of each respective superordinate category.

Significantly stronger negative moods were reported by female teachers (*M* = 32.61, *SE* = 0.53; possible value range: 11–66) compared with their male counterparts (*M* = 30.53, *SE* = 0.83). The intensity of negative affect significantly varied also between single individuals (*M* = 34.02, *SE* = 0.86) and teachers having partners or families (*M* = 31.50, *SE* = 0.53), with the former group feeling more negative emotions than the latter. The differences in negative affectivity were also age-specific; however, they were unrelated to teachers’ professional experience.

The observed differences with respect to gender, relationship status, age and length of professional experience seem to suggest that teachers’ negative affect during the COVID-19 pandemic may be related not only to psychological dispositions and states, but also to particular sociodemographic and situational variables.

To probe which of the factors affecting professional adaptation to emergency remote instruction are associated with teachers’ negative affect and to answer **RQ2**, we conducted correlation analyses (Table 3). Negative affect was significantly and positively correlated with higher situational anxiety (*r* = 0.47) and situational loneliness (*r* = 0.36). Moreover, the stronger the negative emotional state, the less the teachers reported work-life synergy (*r* = –0.43) and the less productive they felt (*r* = –0.33) during the pandemic. Finally, those who felt more negative emotions were also coping worse than others (*r* = –0.30).

**TABLE 3:** Pearson’s *r*^[1]^ and Spearman’s ρ^[2]^ correlation coefficients between negative affect and factors influencing professional adaptation to emergency remote instruction in the COVID-19-related context.

	Correlation with negative affect	*R*^2^	95%CI	
Situational anxiety^1^	0.47*	0.22	0.17	0.29
Work-life synergy^1^	−0.43*	0.19	0.14	0.24
Situational loneliness^1^	0.36*	0.13	0.09	0.17
Self-productivity^2^	−0.33*	0.11	0.07	0.15
Situational coping^2^	−0.30*	0.10	0.06	0.14
Age^1^	–0.27	0.07	0.04	0.10
Family and social support^1^	–0.18	0.03	0.01	0.05
Professional experience^1^	–0.17	0.03	0.01	0.05
Relationship status^2^	0.15	0.02	0.00	0.04
Gender^1^	0.05	0.003	–0.004	0.01
Living conditions^2^	–0.02	0	–	–

* significant at *p* < 0.05.

To reveal which of the factors affecting professional adaptation to emergency remote instruction predict teachers’ negative affect, as well as provide an answer to RQ3 concerning the relative contribution of the respective predictors and the extent to which each of them determines teachers’ negative affect and, consequently, emotional wellbeing, we used ANCOVA to build a stepwise regression model with the forward selection procedure. It was preceded by a simple linear regression giving more general insight into the extent to which all the factors affecting professional adaptation to emergency remote instruction predict teachers’ negative affect. The entire model, as shown in Table 4, is significant (*F*_8,__317_ = 37.40, *p* < 0.001) and predicts approximately 49% of variance in negative affect. The most influential predictor of teachers’ negative emotional states was anxiety (β = 0.30, *t* = 6.82, *p* < 0.001), explaining approximately 22% of the variance in negative affect. The subsequent most consequential predictors turn out to be work-life synergy (β = 0.25. *t* = –5.81. *p* < 0.001) followed by teacher productivity (reduced: β = 0.26, *t* = 5.62, *p* < 0.001; unchanged: β = –0.15, *t* = –3.50, *p* < 0.001). The next important predictor is situational coping (β = 0.26, *t* = 3.16, *p* < 0.001). The last two moderator variables are teachers’ age (β = –0.16, *t* = –3.77, *p* < 0.001) and situational loneliness (β = 0.09, *t* = 2.04, *p* < 0.001). It should be noted that all the influences of these predictors have medium to large effects (Cohen, 1988). Interestingly, family and social support as well as variables such as gender, relationship status, and length of professional experience no longer contribute to the regression model. The results are presented in Table 5.

**TABLE 4:** The regression results of the effects of variables predicting teachers’ negative affect.

Dependent variable	*R*^2^	Adj. *R*^2^	*F*	df1	df2	95%CI	
Negative affect	0.49	0.47	37.40	8	317	0.41	0.53

**TABLE 5:** General linear model with ANCOVA (forward selection stepwise regression) for variables predicting teachers’ negative affect.

Step	Independent variables	b	SE	β	*t*	η_*p*_^2^	95%CI		*F*
1	Situational anxiety	0.64	0.09	0.30*	6.82	0.54	0.47	0.58	46.58
2	Work-life synergy	–2.24	0.38	−0.25*	–5.81	0.46	0.38	0.50	33.71
3	Productivity:					0.30	0.22	0.35	16.98
	reduced	4.31	0.77	0.26*	5.62				
	equal	–2.42	0.70	−0.15*	–3.50				
4	Coping: worse than others	5.75	1.82	0.26*	3.16	0.17	0.09	0.21	8.14
5	Age	–0.17	0.05	−0.16*	–3.77	0.26	0.18	0.31	14.21
6	Situational loneliness	0.33	0.16	0.09*	2.04	0.10	0.03	0.13	4.16
-	Family and social support								
-	Gender								
-	Relationship status								
-	Living conditions								
-	Professional experience								

b, unstandardized regression coefficient; SE, standard errors; β, standardized regression coefficient; * significant at p < 0.001.

## Discussion and Conclusion

The current COVID-19 pandemic is unprecedented in scale, as are the nearly global school closures that have gone along with it. The well-being of students and instructors has been upended, to the extent that many instructors started contemplating quitting the profession (Business Wire, 2021; Gewin, 2021). The current study has attempted to reveal some of the mechanisms responsible for college and university instructors’ differential coping with the transition and wellness, with the resultant model predicting approximately 49% of variance in negative affect.

The impact of anxiety was prominent not only in the statistics, but was also a recurrent theme in the open-ended questions, where the respondents repeatedly mentioned “anxiety and fear about the pandemic,” “general anxiety about the world right now,” “anxiety, workload, uncertainty,” “my own personal struggle with anxiety increasing due to covid,” and “the uncertainty and anxiety surrounding the state of the world in general.”

The impact of gender on anxiety (albeit with only a very small effect size) mirrors the findings by Alemany-Arrebola et al. (2020) among college students, where women had higher scores in trait and state anxiety; the latter accentuated in cases where a relative had died – thus showing that the stressful situation of the pandemic and remote learning together with a critical event such as the illness and death of a relative or friend due to COVID-19 increases anxiety levels (and—in the case of the original study—influences the perception of academic self-efficacy). A similar conclusion was reported in Turkey by Aslan et al. (2020), where female (as well as less physically active) students displayed higher levels of perceived stress and by Karaman et al. (2021), in Switzerland by Amendola et al. (2021), and in the US (Business Wire, 2021; Clabaugh et al., 2021), while among teachers a similar trend was observed in the Basque Country (Ozamiz-Etxebarria et al., 2021). In the general population, three studies carried out in Italy (Mazza et al., 2020; Moccia et al., 2020) and Iran (Moghanibashi-Mansourieh, 2020) showed women’s mental health to be more impacted than men’s; on the other hand, research in China (Cao et al., 2020; Chen et al., 2020; Huang and Zhao, 2021) and a meta-analysis by Cénat et al. (2021a) of papers again mainly hailing from this country found no gender differences in females’ and males’ experience of pandemic-related stressors. A comparison of the results implies a possible role of context (teaching/studying vs others) influencing the potential role of gender in perceived situational anxiety.

Apprehension about job stability could be alleviated by institutions making furlough arrangements for all faculty and suspensions of staff dismissals at least during the period of the crisis, in line with recommendations by the World Bank, since “faculty dismissed at this time will find it almost impossible to secure alternative employment amid the crisis” (2020:10).

The adverse impact of loneliness is fairly straightforward and, as with anxiety, was a recurrent theme in the answers to the open-ended questions, which frequently mentioned “isolation, not being able to interact with my students, not being able to teach them;” “I feel isolated. The teaching is fine, but I have had very little interaction with colleagues, which makes me feel irrelevant;” “lack of contact with the students. I felt very isolated and like I was talking into the void;” “the isolation and feeling that I am not connecting with students and fellow teachers in my school;” or “disconnection from students.” Loneliness constitutes an emotional experience provoked by unfulfilled needs for social contact (Margalit, 2010). Cacioppo and Hawkley (2009) point out that the experience of loneliness impairs individual ability to self-regulate affecting physiological, cognitive and emotional functioning. It may constitute an important risk factor for depressive symptoms even for healthy people (Cacioppo et al., 2006; Grov et al., 2010). Neto and Barros (1992) in their study examining loneliness among teachers found that educators whose professional experience was longer than 20 years were significantly lonelier than those with a shorter professional experience; the former group also experienced lowered self-efficacy, which constitutes one of the predictors of job satisfaction (Lent and Brown, 2006; Duffy and Lent, 2009). While loneliness is usually a temporary state, COVID-19-induced lockdown and social distancing may have extended it. Lack of personal contact with teachers and longing for live communication during the lesson were downsides of remote instruction indicated by around 80% of the students in a study by Karalis and Raikou (2020).

The significance of the right work-life synergy and maintaining the right priorities is especially important in view of the reality that ensuring of teacher and student well-being may sometimes be at odds with pressures to “cover the syllabus”: “[t]here is a potential tradeoff between ensuring well-being and significantly increased screen time derived from a transition to distance learning” (Reimers and Schleicher, 2020:8). Our findings from a sample of nearly 1,500 educators (Jelińska and Paradowski, 2021a) showed that the most engaged and best-coping teachers were characterized by having modified their lesson plans and eased the grading scheme during this period.

Teacher well-being should never be forgotten, neither by administrators nor teachers themselves. Already in the spring of 2020, a guide published by the OECD recommended that:

*Second only to supporting learning, a key priority of education institutions should be the well-being of students and staff*… *A protracted pandemic, and its multiple effects in the health, income and well-being of individuals and communities, is likely to strain the psychological reserves of all, including students and teachers. Educators and leaders of education systems should make explicit and visible their goals for well-being, and pursue strategies that help maintain well-being in the face of a global health event that will have a considerable toll in the lives and health of individuals*… *As such impact becomes proximal to every learner and educator, this may impact their motivation and functioning.* (Reimers and Schleicher, 2020:7*f*).

At the time of writing this paper, a discussion is ranging in the press and on teacher groups in the social media concerning the coverage of a story where a teacher has been giving lessons from her hospital bed following a surgery. While many news outlets were portraying the action as a model and inspiration, most teachers have rightly expressed frustration at the unhealthy, dystopian expectations of a broken education system. Teachers deserve a humanistic approach, especially during the added strain of emergency remote instruction. Our respondents commented: “Thank you for sharing this survey! I enjoyed completing it, and thought it was one of the more comprehensive and compassionate surveys I’ve done during this time.”; “Just completed this survey and I too would HIGHLY recommend that you spend some time completing it. It’s LONG as surveys go, so grab yourself a good cup of tea/coffee and some nibbles and find yourself a quiet space, it’s going take over half an hour, but I can safely say I have never felt that a survey could be life affirming but this is! It actually made me feel better. It’s like your own personal therapist at these difficult times. Thank you! Well done.” and “I was able to relate all the questions to my experiences. I appreciated that the survey seemed to focus on the educator as a whole person rather than just a ‘provider of lessons and materials.’,” underlining the importance of seeing teachers in a holistic and humanistic manner.

Despite the challenges of emergency remote teaching, HE instructors are in a privileged position compared with most of their counterparts teaching at lower levels of education, as the education level handled has already turned out to be a significant predictor of coping and engagement (Jelińska and Paradowski, 2021a).

The post-pandemic world may see the trend of classes becoming more blended (Bozkurt, 2019; Kim, 2020), integrating conventional face-to-face instruction with online learning (Garrison and Kanuka, 2004; Dziuban et al., 2018). The “new normal” will hopefully remove most of the current stressors, but awareness of the variables influencing teacher coping and well-being will be nonetheless be useful to both teachers and administrators.

One of the obvious limitations, shared by most large-scale surveys, is the issue of participant self-selection. Given that participation in this study was completely voluntary, and that on many occasions the questionnaire took upwards of 45 min to complete, the respondents were already motivated, could relate to the topic, and had the spare time and technology to comfortably fill it out. This means a limit on the representativeness and generalization potential of the data and resultant findings (see Brown, 2001:85).

This university instructors’ perspective will be complemented with later publications on other relevant aspects of educators’ adaptation to the transition, as well as analyses of students’ points of view.

## Data Availability Statement

The data analyzed in this study is subject to the following licenses/restrictions: The data are part of a larger project still in progress. Requests to access these datasets should be directed to MBP, https://schoolclosure.ils.uw.edu.pl.

## Ethics Statement

The survey protocol had been reviewed and approved by University of Warsaw’s Human Research Ethics Committee. The respondents were provided with information about the survey and participated voluntarily.

## Author Contributions

MBP conceptualized the study, designed the survey instrument, piloted and administered the data collection, and contributed to the interpretation of the data and the writing of the article. MJ contributed to the survey instrument, carried out the quantitative analyses, and contributed to the writing of the article. Both authors approved the submitted version.

## Conflict of Interest

The authors declare that the research was conducted in the absence of any commercial or financial relationships that could be construed as a potential conflict of interest.

## Footnotes

1 The data analysed in this paper form part of a much larger project https://schoolclosure.ils.uw.edu.pl that also included educators teaching in the K–12 track as well as student populations. These latter data are omitted from the analyses herein. For reasons of space and thematic coherence, we also exclude a presentation and discussion of other contextual findings as well as those from the battery of psychological tests that concluded the questionnaire.
