# Supplementary material for: The Impact of Demographics, Life and Work Circumstances on College and University Instructors’ Well-Being During Quaranteaching
Source: Front Psychol. 2021 Jun 11;12:643229. doi: 10.3389/fpsyg.2021.643229 (PMC8226323; doi:10.3389/fpsyg.2021.643229)
Supplement: Supplementary file 1 [file Data_Sheet_1.pdf]

Jelińska, M. and Paradowski, M. B. (2021). The impact of demographics, life and work circumstances on college and university instructors' well-being during quaranteaching: Findings from a global survey. Frontiers in Psychology, 12, 643229 [Research topic "Covid-19 and Beyond: From (Forced) Remote Teaching and Learning to 'The New Normal' in Higher Education"]. DOI: 10.3389/fpsyg.2021.643229. Supplementary Material: survey.

Educators' adaptation to remote instruction during the COVID-19 epidemic

Page description:
Welcome to this survey being carried out by a team of researchers at the University of Warsaw! We intend to find out how teachers have been coping with the **switch to remote instruction** during the COVID-19 epidemic. Specifically, we hope to better understand what circumstances, behaviours, attitudes and psychological traits make it easier to deal with the new reality, what challenges you have been facing, and what has helped you and your well-being. We believe you will relate to many of the questions here.
The survey has research (IRB) clearance, is anonymous and should take from 25 to upwards of 45 minutes to complete, depending on the detailedness of your answers. You may abandon this survey at any time. Partial responses are saved as well, but only up to the last submitted page.
Your answers will only be used for scientific research and won't be used for any commercial purposes. No information is collected that could identify your school. The results will be used in articles that will be submitted to pedagogy journals and books. If you want to know the results of the survey, you have the option to leave an e-mail address at the end. The e-mail will be doubly password-protected on a secure platform; after maximum one month it will be removed and securely stored offline in a password-protected file. Moreover, you will have the option to leave an e-mail address at the end if you agree to also take part in an optional 12-15-minute second stage of the survey once remote teaching has ended. In that case the e-mail will be used to send you a unique link to the follow-up survey; afterwards, to preserve complete anonymity the e-mail address will be removed from the database and replaced with a number.
You will answer questions about the circumstances surrounding your transition to remote instruction, your personal experiences, behaviours, attitudes, feelings, physical and mental health, and your personality traits. There are no specific risks involved in taking part in the survey. Some of the questions about your experiences during this period may be difficult for anxiety sufferers.
Once you have completed the survey, your answer will be saved in the dataset. If you decide to withdraw after completing this questionnaire, you may communicate this intention by writing to the lead researcher, Dr Michał B. Paradowski, at michal[dot]paradowski[-AT-]uw[dot]edu[dot]pl, citing the unique survey ID number that will be visible in your survey's URL (in the address bar of your browser). Your answers will then be deleted at the earliest possible time.
If you have any questions, you may contact the lead researcher, Dr Michał B. Paradowski, at michal[dot]paradowski[-AT-]uw[dot]edu[dot]pl. Thank you in advance for honest responses!

This is a survey for **EDUCATORS in subjects other than language**. We are also running parallel but different, specific surveys:

for LANGUAGE TEACHERS at xxxxxxxxxxxxxxxxxxxxxxxxxxxxxxxxxxxxxxxxxxxxxxxxxxxxxxxxxxxxxxx
for LANGUAGE LEARNERS at xxxxxxxxxxxxxxxxxxxxxxxxxxxxxxxxxxxxxxxxxxxxxxxxxxxxxxxxxxxxxxx
for LINGUISTICS INSTRUCTORS AND TAs at xxxxxxxxxxxxxxxxxxxxxxxxxxxxxxxxxxxxxxxxxxxxxxxxxxxxxxxxxxxxxxx
for LINGUISTICS STUDENTS at xxxxxxxxxxxxxxxxxxxxxxxxxxxxxxxxxxxxxxxxxxxxxxxxxxxxxxxxxxxxxxx
and a Polish-language version of this survey at xxxxxxxxxxxxxxxxxxxxxxxxxxxxxxxxxxxxxxxxxxxxxxxxxxxxxxxxxxxxxxx

If you know colleagues, students or friends who qualify and might be willing to fill in any of the surveys, please feel free to forward the relevant link(s). Thank you!

1. Do you agree to take part in this survey? *

- Yes

2. Have you had to transition from teaching regular face-to-face classes to teaching remotely (online) as part of the response to the COVID-19 epidemic? *

- Yes
- No

3. Country you are teaching in now: *

4. Is this your home country?

- Yes
- No

5. Age

6. Gender *

- Male
- Female
- Non-binary/Not listed

7. I have a degree in

Jelińska, M. and Paradowski, M. B. (2021). The impact of demographics, life and work circumstances on college and university instructors' well-being during quaranteaching: Findings from a global survey. Frontiers in Psychology, 12, 643229 [Research topic "Covid-19 and Beyond: From (Forced) Remote Teaching and Learning to 'The New Normal' in Higher Education"]. DOI: 10.3389/fpsyg.2021.643229. Supplementary Material: survey.

8. In what type of school are you currently teaching? *

- ☐ preschool/kindergarten
- ☐ elementary/primary school
- ☐ middle/junior high school
- ☐ secondary/high school
- ☐ vocational school
- ☐ community college/college/undergraduate school
- ☐ university/graduate school
- ☐ university of applied sciences
- ☐ teacher training college
- ☐ private tuition/self-employed/freelance
- ☐ other (please specify):

9. Is it a

state school
private shool
religious school
school run by an NGO/foundation

10. Languages you speak and your level of competence. Please list all you speak, together with the level, choosing from "Native", "A1" (≈beginner), "A2" (≈pre-intermediate), "B1" (≈intermediate), "B2" (≈upper-intermediate), "C1" (≈advanced), "C2" (≈native-like). For example: *Korean – native; English – B2; Polish – A1*. You will find a brief reference scale below. *

Explanation of the levels:

Native: The language(s) you have been brought up in and are fluent in.
A1: I can use simple phrases and sentences to describe where I live and people I know.
A2: I can use a series of phrases and sentences to describe in simple terms my family and other people, living conditions, educational background and present or last job.
B1: I can connect phrases in a simple way in order to describe experiences and events, my dreams, hopes and ambitions. I can briefly give reasons and explanations for opinions and plans. I can narrate a story or the plot of a book or film and describe my reactions.
B2: I can present clear, detailed descriptions on a wide range of subjects related to my interests. I can explain a viewpoint giving the advantages and disadvantages of various options.
C1: I can present clear, detailed descriptions of complex subjects, developing particular points and rounding off with an appropriate conclusion.
C2: I can present a clear, smoothly flowing description or argument in a style appropriate to the context and with an effective logical structure which helps the listener/reader notice and remember significant points.

11. What subject(s) have you been teaching remotely this semester/trimester (if applicable)?

12. I had full freedom deciding on the topic and content of this/these course(s). *

1 - Completely disagree
2 - Mostly disagree
3 - Slightly disagree
4 - Somewhat agree
5 - Mostly agree
6 - Completely agree

13. I have been teaching this subject for * ______ years

14. How long is each class (during the pandemic)? * ______ minutes

15. How many times do you meet per week remotely? *

Jelińska, M. and Paradowski, M. B. (2021). The impact of demographics, life and work circumstances on college and university instructors' well-being during quaranteaching: Findings from a global survey. Frontiers in Psychology, 12, 643229 [Research topic "Covid-19 and Beyond: From (Forced) Remote Teaching and Learning to 'The New Normal' in Higher Education"]. DOI: 10.3389/fpsyg.2021.643229. Supplementary Material: survey.

16. What size is the (average) class you are teaching? *

[ ] students

17. What is the average age of the students in your class?

approximately [ ] years

18. What percentage of the class (if any) are international students? *

around [ ] %

19. Some of my current students are now in different time zones. *

- ○ Yes
- ○ No

20. What % of your students have not been taking part in the remote classes?

around [ ] %

21. Before the epidemic, I used to spend around *

[ ] hours at the school per week (physically).

22. I am

- ○ single
- ○ in a relationship

23. Currently, I am living *

- ○ on my own
- ○ with my parents (and siblings)
- ○ with my partner
- ○ with my partner and children
- ○ with my children
- ○ with my family including children
- ○ with my (family including) parents
- ○ with my partner and my or her/his parents
- ○ with my parents and children (multigenerational family)
- ○ with (a) roommate(s)/flatmate(s)/sibling(s)

24. I come from a:

- village
- town under 10,000 inhabitants
- town between 10,000 and 100,000 inhabitants
- town between 100,000 and 500,000 inhabitants
- town/city between 500,000 and 1 million inhabitants
- city of more than 1 million people

25. What are your current teaching conditions? (select all that apply) *

- ☐ I have my own room where I can teach online undisturbed.
- ☐ I am currently living in the same location where I normally teach/live.
- ☐ I am currently living in a different place from where I normally live.
- ☐ I have to look after my children or other relatives.
- ☐ I have been involuntarily separated from my family/partner.
- ☐ I have another job apart from teaching.

26. How long have you been teaching remotely now since the transition as part of a response to the epidemic? *

since [ ] �

27. Have regular (face-to-face) classes resumed at your school yet? *

- Yes
- No

28. Has the teaching semester/trimester ended (if applicable)? *

- Yes
- No

29. When is the end of the semester? *

�

30. Have you received training in online/remote teaching? (select all that apply) *

- No training
- Self-training with online resources (videos, webinars, articles, Facebook support groups, etc.)
- Occasional/informal consultation with a colleague
- Training with an instructor before the switch to remote teaching
- Training or workshops in remote/online teaching on multiple occasions before the epidemic
- I had given training in remote/online teaching to other teachers
- Other (please specify):

31. I had been teaching e-learning, blended courses, MOOCs, or webinars before this epidemic. *

- Yes
- No

32. I had taught a flipped classroom before this epidemic (students review course materials at home, and spend in-class time on problem sets, discussions and independent work under my guidance). *

- Yes
- No

33. How often did you use interactive teaching tools in normal (non-remote) teaching before the epidemic?

- never
- occasionally
- frequently
- almost every lesson

34. What teaching method(s) are you using now? *

- I teach classes remotely in real time (live, synchronous classes).
- I've been using a flipped classroom approach (providing materials in advance, and spending actual interactive class time working with the students on the problem sets).
- I prerecord my lectures and upload them online (with a forum for questions).
- I record the synchronous classes and then make them available online for later viewing.
- I only post materials for the students online and grade their assignments later, but I do not teach in real time or prerecord lectures.

35. Please specify why: *

Jelińska, M. and Paradowski, M. B. (2021). The impact of demographics, life and work circumstances on college and university instructors' well-being during quaranteaching: Findings from a global survey. Frontiers in Psychology, 12, 643229 [Research topic "Covid-19 and Beyond: From (Forced) Remote Teaching and Learning to 'The New Normal' in Higher Education"]. DOI: 10.3389/fpsyg.2021.643229. Supplementary Material: survey.

36. Do you like the tools/software/platform you have been using to teach the online classes? *

1 - I hate it!
2
3
4
5
6 - I love it!

37. I access the classes on my *

- ☐ computer/laptop
- ☐ tablet/iPad
- ☐ phone

38. How much time does it take you to prepare one remote class in your present course(s)? *

around [ ] hours

39. That is around *

[ ] % of the time it would normally take me in the case of a face-to-face course.

40. Why?

[ ]

41. I had *

[ ] days to prepare for the transition to remote teaching.

42. To what extent do you agree with the following: *

	completely disagree	mostly disagree	slightly disagree	somewhat agree	mostly agree	completely agree
That was not enough time to get sufficiently familiar with remote/online teaching tools.	○	○	○	○	○	○
I feel disengaged in the remote teaching.	○	○	○	○	○	○
I am willing to devote more time to an online class.	○	○	○	○	○	○
I have been trying my best to teach well during this time.	○	○	○	○	○	○
My course(s) is/are <u>not</u> my highest priority right now.	○	○	○	○	○	○
I want this remote teaching to be over and to be back in a regular class!	○	○	○	○	○	○
I hadn't expected remote teaching to be this hard.	○	○	○	○	○	○
I preferred the 'normal' class format to the remote one I'm teaching now.	○	○	○	○	○	○
My institution was unprepared for this situation.	○	○	○	○	○	○
I felt confident in my ability to teach remotely when I was told to do so.	○	○	○	○	○	○
I have someone to turn to for support if I experience technical problems with the remote teaching.	○	○	○	○	○	○
I had full freedom in how to adapt my course(s).	○	○	○	○	○	○
At least I am not wasting time on commuting to the school.	○	○	○	○	○	○
I feel that I have been left behind by the shift to remote teaching.	○	○	○	○	○	○
I feel that some of my students have been left behind/fallen through the cracks in the shift to remote teaching.	○	○	○	○	○	○
My students have not coped well with remote learning.	○	○	○	○	○	○
My students have responded positively to my remote teaching.	○	○	○	○	○	○
Thanks to the change, some students who used to be passive have now become more active.	○	○	○	○	○	○
The class is calmer and easier to control now.	○	○	○	○	○	○
Now my students ought to be able to do more because they have more time.	○	○	○	○	○	○
I have been trying to use this opportunity to make my classes inclusive and accessible to everyone, in line with universal design (UDL/508 compliant, e.g. [illegible]	○	○	○	○	○	○

design (UDL/508 compliant, e.g. accessible for students with disabilities).						
I have had to modify my lesson plans for remote teaching.	○	○	○	○	○	○
I have eased the grading scheme.	○	○	○	○	○	○
During this epidemic, I have felt that I have to alter not just the medium and method, but also the content of my classes.	○	○	○	○	○	○

43. How?

44. I find that during the remote teaching, the classes are *

- more efficient
- equally efficient
- less efficient

45. I think the current pandemic situation is affecting *

- me more than the students
- me and students equally
- the students more than me

46. I use a virtual background in the video teaching.

- ○ Yes
- ○ No
- ○ Not applicable – I am not teaching live (synchronous) classes

47. Why?

48. Since your school was closed because of the coronavirus, how often have you been interacting with the majority of your students? *

around ______ times per week.

49. To what extent do you agree with the following: *

	completely disagree	mostly disagree	slightly disagree	somewhat agree	mostly agree	completely agree
It bothers me not being able to see my students' reactions.	○	○	○	○	○	○
I try to stay in touch with my colleagues every day.	○	○	○	○	○	○
I miss daily conversations with my colleagues.	○	○	○	○	○	○
I try to reassure my students during these times.	○	○	○	○	○	○
Teaching remotely, I feel like I'm speaking to a brick wall.	○	○	○	○	○	○
I often ask students to split into pairs/ groups/ breakout rooms during the online class.	○	○	○	○	○	○

50. What has helped you cope with the transition to remote teaching? *

Jelińska, M. and Paradowski, M. B. (2021). The impact of demographics, life and work circumstances on college and university instructors' well-being during quaranteaching: Findings from a global survey. Frontiers in Psychology, 12, 643229 [Research topic "Covid-19 and Beyond: From (Forced) Remote Teaching and Learning to 'The New Normal' in Higher Education"]. DOI: 10.3389/fpsyg.2021.643229. Supplementary Material: survey.

51. What has made it difficult for you to transition to remote teaching? *

52. Have you experienced any of these problems while teaching remotely? *

	completely disagree	mostly disagree	slightly disagree	somewhat agree	mostly agree	completely agree
I have often encountered technical problems with the class (unreliable Internet connection, audio/video issues, etc.).	○	○	○	○	○	○
I haven't been able to fully utilise the potential of remote teaching because of lack of access to hardware (computer/ webcam/tablet/printer/scanner …).	○	○	○	○	○	○
My computer is too slow for the remote teaching software.	○	○	○	○	○	○
I lack access to printed materials and resources to prepare my classes.	○	○	○	○	○	○
I worry about privacy and the security of the software/platform.	○	○	○	○	○	○
I am a bit uneasy about the possibility of my classes being seen by people other than only my students.	○	○	○	○	○	○
My class has been crashed or 'bombed' by unauthorised participants.	○	○	○	○	○	○
I find it difficult to evaluate students' activity during online classes.	○	○	○	○	○	○
I am unable to verify whether the students are learning.	○	○	○	○	○	○
I am anxious that some students may be using the situation to cheat in their assignments/exams.	○	○	○	○	○	○
The students have encountered technical problems with the class (unreliable Internet connection, audio/video issues, etc.).	○	○	○	○	○	○
I feel that remote teaching reduces my possibility to meet students' individual/ special needs.	○	○	○	○	○	○
The students have trouble with concentration and staying focused during the online classes.	○	○	○	○	○	○

53. Some of my students need to share their computer with household members. *

Yes
No
I don't know

54. To what extent do you agree with the following: *

	completely disagree	mostly disagree	slightly disagree	somewhat agree	mostly agree	completely agree
I try to keep a daily routine.	○	○	○	○	○	○
I can decide freely whether I can leave the house or not.	○	○	○	○	○	○
I check updates/news on COVID-19 every day.	○	○	○	○	○	○
I have no control over whether I will catch the virus.	○	○	○	○	○	○
I am exposed to the virus via my family member(s)/flatmate(s)/co-worker(s)/ customers…	○	○	○	○	○	○
I am at high risk of severe illness from COVID.	○	○	○	○	○	○
My close family member/partner is at high risk of severe illness from COVID.	○	○	○	○	○	○
Things will get back to normal.	○	○	○	○	○	○
I will come out of this unscathed.	○	○	○	○	○	○
The epidemic has brought about some positive developments.	○	○	○	○	○	○
If I and my family stay healthy and survive the epidemic, I will consider it a success.	○	○	○	○	○	○
Once this is over, at the very least I will have learnt some new tech skills and methods.	○	○	○	○	○	○
I feel anxious about my future.	○	○	○	○	○	○
I worry about my job stability.	○	○	○	○	○	○
I worry about housing instability.	○	○	○	○	○	○
I worry about my family/friends.	○	○	○	○	○	○
I worry about the future of my community/ society.	○	○	○	○	○	○
I worry about climate change.	○	○	○	○	○	○
The current crisis will further social inequalities.	○	○	○	○	○	○
I am afraid of an approaching economic crisis.	○	○	○	○	○	○

Jelińska, M. and Paradowski, M. B. (2021). The impact of demographics, life and work circumstances on college and university instructors' well-being during quaranteaching: Findings from a global survey. Frontiers in Psychology, 12, 643229 [Research topic "Covid-19 and Beyond: From (Forced) Remote Teaching and Learning to 'The New Normal' in Higher Education"]. DOI: 10.3389/fpsyg.2021.643229. Supplementary Material: survey.

55. After this epidemic is over, my life is going to *

- change for the better compared with the time before the epidemic
- stay the same
- change for the worse compared with the time before the epidemic

56. When do you hope the lockdown will be over: *

- early May
- mid-May
- late May
- early June
- mid-June
- late June
- early July
- late July
- early August
- late August
- September
- October
- November
- later

57. My social life has been reduced by around *

____ %

58. Are the following situations relevant for you? *

	Yes	No
I have had to buy new hardware and/or a better Internet data plan to be able to teach online.	○	○
I keep going to work.	○	○
I have lost my job due to the epidemic.	○	○
I have been furloughed (put on leave) due to the epidemic.	○	○
A close friend, partner, or family member has lost her/his job or gone bankrupt due to the epidemic.	○	○
I had/have difficulty returning home or to the school due to travel (e.g. flight) disruptions.	○	○
I had/have difficulty returning home or to the school due to an immigration or visa ban.	○	○
I caught COVID-19.	○	○
I have a close friend/family member who caught COVID-19.	○	○
I have a close friend/family member who died after catching COVID-19.	○	○

59. To what extent do you agree with the following: *

	completely disagree	mostly disagree	slightly disagree	somewhat agree	mostly agree	completely agree
I have a good work ethic.	○	○	○	○	○	○
I have been keeping a regular sleep routine.	○	○	○	○	○	○
I have been getting spine-/backache from all the sitting in front of the computer.	○	○	○	○	○	○
My eyes have been aching or dry from the prolonged screen time.	○	○	○	○	○	○
My travel plans have been ruined.	○	○	○	○	○	○
I have been unable to make an appointment with a doctor/dentist.	○	○	○	○	○	○
I am missing lost teacher conference trips.	○	○	○	○	○	○
My situation is not the worst.	○	○	○	○	○	○
I have been through crises before.	○	○	○	○	○	○
I prefer to know bad news to uncertainty.	○	○	○	○	○	○

60. I think I have been coping *

- better than others
- same as others
- worse than others

61. During this time I have been sleeping *

- worse than usual
- same as usual
- better than usual

62. To what extent do you agree with the following: *

	completely disagree	mostly disagree	slightly disagree	somewhat agree	mostly agree	completely agree	**not applicable**
I enjoy it when my students are joined by their family members or roommates when we're having an online class.	○	○	○	○	○	○	○
Most of my students have their family's support.	○	○	○	○	○	○	○
I feel the support of my family/ flatmates/ friends/community during this time.	○	○	○	○	○	○	○
I feel supported by my institution.	○	○	○	○	○	○	○
I feel comfortable having my family/ partner/roommates/flatmates around during this time.	○	○	○	○	○	○	○
I have good relations at home.	○	○	○	○	○	○	○
I am very different from my family/ flatmates where I am staying now.	○	○	○	○	○	○	○
I enjoy being able to spend more time with my family/children/ partner.	○	○	○	○	○	○	○
I prefer to go shopping myself to protect my family's/partner's/ flatmates' health.	○	○	○	○	○	○	○

Jelińska, M. and Paradowski, M. B. (2021). The impact of demographics, life and work circumstances on college and university instructors' well-being during quaranteaching: Findings from a global survey. Frontiers in Psychology, 12, 643229 [Research topic "Covid-19 and Beyond: From (Forced) Remote Teaching and Learning to 'The New Normal' in Higher Education"]. DOI: 10.3389/fpsyg.2021.643229. Supplementary Material: survey.

63. To what extent do you agree with the following: *

	completely disagree	mostly disagree	slightly disagree	somewhat agree	mostly agree	completely agree
I (have volunteered to) help others during these times.	○	○	○	○	○	○
These days I am more responsive to others' needs.	○	○	○	○	○	○
I avoid helping others to protect myself.	○	○	○	○	○	○
My daily life is lacking structure.	○	○	○	○	○	○
I have been taking it easy and slowing down during this time.	○	○	○	○	○	○
I feel I've been wasting my time.	○	○	○	○	○	○
I manage to maintain a work-life balance.	○	○	○	○	○	○
I try to disconnect/keep offline hours.	○	○	○	○	○	○
I finally have more free time.	○	○	○	○	○	○
I feel enthusiastic about this opportunity to learn new skills.	○	○	○	○	○	○
I have started learning another language.	○	○	○	○	○	○
I have been using the time to read books or watch films that I had no time for earlier.	○	○	○	○	○	○
I have been binge-watching.	○	○	○	○	○	○
I have been visiting cultural institutions online (virtual tours of museums/galleries, online retransmissions of theatre plays/ music concerts, etc.).	○	○	○	○	○	○
I have been spending more time on creative activities and hobbies (cooking/baking/ BBQing/gardening/ playing music/painting…).	○	○	○	○	○	○
I keep physically active (yoga/treadmill/ bicycle/running/Pilates…).	○	○	○	○	○	○
I have been spending more time in nature.	○	○	○	○	○	○
I go out to get fresh air every day.	○	○	○	○	○	○
I have been spending more time on social media.	○	○	○	○	○	○
I have found new inspiration during this time.	○	○	○	○	○	○
I have reassessed my value system.	○	○	○	○	○	○

64. How have you been feeling during the lockdown restrictions? *

	completely disagree	mostly disagree	slightly disagree	somewhat agree	mostly agree	completely agree
I am watching this epidemic unfold as an interesting experiment.	○	○	○	○	○	○
Finally, there is something exciting going on!	○	○	○	○	○	○
My dreams have been more emotional/ anxious.	○	○	○	○	○	○
I feel I have been losing my sanity.	○	○	○	○	○	○
I find it harder to get out of bed in the morning.	○	○	○	○	○	○
I feel tired during the day.	○	○	○	○	○	○
I am feeling exhausted all the time.	○	○	○	○	○	○
I have been feeling irritable.	○	○	○	○	○	○
I feel building up pressure.	○	○	○	○	○	○
I have been sad.	○	○	○	○	○	○
I have been having bouts of anxiety/panic attacks.	○	○	○	○	○	○
I feel no motivation to do anything.	○	○	○	○	○	○
I am afraid to leave my home.	○	○	○	○	○	○
I feel lonely.	○	○	○	○	○	○
I have been enjoying the solitude.	○	○	○	○	○	○
I used to pretend caring about meaningless things at work that now I finally don't have to.	○	○	○	○	○	○
I have been kind to myself.	○	○	○	○	○	○
It's okay to feel the way I'm feeling these days.	○	○	○	○	○	○
I think that during these times, my teaching does not need to be perfect. If it is "good enough," that will do.	○	○	○	○	○	○

Jelińska, M. and Paradowski, M. B. (2021). The impact of demographics, life and work circumstances on college and university instructors' well-being during quaranteaching: Findings from a global survey. Frontiers in Psychology, 12, 643229 [Research topic "Covid-19 and Beyond: From (Forced) Remote Teaching and Learning to 'The New Normal' in Higher Education"]. DOI: 10.3389/fpsyg.2021.643229. Supplementary Material: survey.

65. I have been ________ productive than before. *

- more
- equally
- less

66. The questions in this short section ask about your feelings and thoughts during the last two weeks. In each case, please indicate how often you felt or thought a certain way.

In the last two weeks: *

	Never	Almost never	Sometimes	Fairly often	Very often
… how often have you been upset because of the lockdown restrictions and the remote teaching?	○	○	○	○	○
… how often have you felt that you were unable to control the important things in your life?	○	○	○	○	○
… how often have you felt nervous and stressed?	○	○	○	○	○
… how often have you felt confident about your ability to handle the situation?	○	○	○	○	○
… how often have you felt that things were going your way?	○	○	○	○	○
… how often have you found that you could not cope with all the things that you had to do?	○	○	○	○	○
… how often have you been able to control irritations in your life?	○	○	○	○	○
… how often have you felt that you were in control of things?	○	○	○	○	○
… how often have you been angered because of things that were outside of your control?	○	○	○	○	○
… how often have you felt difficulties were piling up so high that you could not overcome them?	○	○	○	○	○

67. **This final section looks at your more general (stable) personality traits and behaviours (part 1/4)** *

	completely disagree	mostly disagree	slightly disagree	somewhat agree	mostly agree	completely agree
By working hard, a person can achieve anything.	○	○	○	○	○	○
I enjoy trying out new teaching methods.	○	○	○	○	○	○
I like to solve complex problems.	○	○	○	○	○	○
I get things done quickly.	○	○	○	○	○	○
I like to leave my comfort zone.	○	○	○	○	○	○
I am usually a patient person.	○	○	○	○	○	○
I easily adapt to new situations.	○	○	○	○	○	○
I like to follow a regular schedule.	○	○	○	○	○	○
My timetable and deadlines are manageable and realistic.	○	○	○	○	○	○
I love my job.	○	○	○	○	○	○
When I fail at something important to me, I become consumed by feelings of inadequacy.	○	○	○	○	○	○
I take responsibility for the way I teach.	○	○	○	○	○	○
When preparing the class, I try to put myself in my students' position.	○	○	○	○	○	○
I can talk my students into learning.	○	○	○	○	○	○
I avoid contacts with others.	○	○	○	○	○	○
I make friends easily.	○	○	○	○	○	○
I like to move around when I'm teaching.	○	○	○	○	○	○
I look at the bright side of life.	○	○	○	○	○	○
I have little influence over the way people behave.	○	○	○	○	○	○
I like the possibility to learn new software.	○	○	○	○	○	○
I try to be understanding and patient towards those aspects of my personality I don't like.	○	○	○	○	○	○
I like to think up new ways of doing things.	○	○	○	○	○	○
I know how to get things done.	○	○	○	○	○	○
I am always the first person ready to act.	○	○	○	○	○	○
I rarely complain.	○	○	○	○	○	○
I am easily annoyed by others' mistakes.	○	○	○	○	○	○
I can adjust my plans to changing	○	○	○	○	○	○

conditions.	○	○	○	○	○	○
I do things according to a plan.	○	○	○	○	○	○
I easily accomplish my work on time.	○	○	○	○	○	○
When something painful happens, I try to take a balanced view of the situation.	○	○	○	○	○	○

68. **(part 2/4)** *

	completely disagree	mostly disagree	slightly disagree	somewhat agree	mostly agree	completely agree
I am good at setting boundaries.	○	○	○	○	○	○
I plunge into tasks with all my heart.	○	○	○	○	○	○
I am not good at taking charge of a group.	○	○	○	○	○	○
I accept people as they are.	○	○	○	○	○	○
I wait for others to lead the way.	○	○	○	○	○	○
I like to have many people around me.	○	○	○	○	○	○
I am willing to talk about myself.	○	○	○	○	○	○
I find it very important to use my body language when I speak.	○	○	○	○	○	○
I will succeed in the goals I set for myself.	○	○	○	○	○	○
I let others determine my choices.	○	○	○	○	○	○
I prefer variety to routine.	○	○	○	○	○	○
I love to hear about other countries and cultures.	○	○	○	○	○	○
I am able to look at problems from a multitude of angles.	○	○	○	○	○	○
Even with difficult tasks, I am always confident.	○	○	○	○	○	○
I tend to face my fears.	○	○	○	○	○	○
When I'm feeling down, I tend to feel most other people are probably happier than I am.	○	○	○	○	○	○
I am not easily frightened.	○	○	○	○	○	○
I can stand criticism.	○	○	○	○	○	○
In an emergency situation, I can put aside feelings and emotions to handle important tasks.	○	○	○	○	○	○
I make a list and try to focus on the most important thing first.	○	○	○	○	○	○
I have good time-management skills.	○	○	○	○	○	○
I take regular breaks and exercise.	○	○	○	○	○	○
I try to see my failings as part of the human condition.	○	○	○	○	○	○
Work is <u>not</u> an important part of my life.	○	○	○	○	○	○
Everyone is responsible for their own success and failures.	○	○	○	○	○	○
I take others' interests into account.	○	○	○	○	○	○
I try to make sure everyone in a group feels included.	○	○	○	○	○	○
I like to talk to other people.	○	○	○	○	○	○
I reveal little about myself.	○	○	○	○	○	○
I find it very important to be able to see my interlocutor's/students' body language and other non-verbal cues.	○	○	○	○	○	○
I look forward to each new day.	○	○	○	○	○	○
How much my students learn depends on me.	○	○	○	○	○	○
I like to visit new places.	○	○	○	○	○	○
When I'm going through a very hard time, I give myself the caring and tenderness I need.	○	○	○	○	○	○

69. **(part 3/4)** *

	completely disagree	mostly disagree	slightly disagree	somewhat agree	mostly agree	completely agree
I like to know how things work.	○	○	○	○	○	○
I like to work on tasks that require a great deal of skill.	○	○	○	○	○	○
Even if something scares me, I will not back down.	○	○	○	○	○	○
I often worry about things that turn out to be unimportant.	○	○	○	○	○	○
It takes a lot to make me feel angry at someone.	○	○	○	○	○	○
I am an open-minded person in dealing with others.	○	○	○	○	○	○
I find it difficult to stick to my plans.	○	○	○	○	○	○
I like order and regularity.	○	○	○	○	○	○

Jelińska, M. and Paradowski, M. B. (2021). The impact of demographics, life and work circumstances on college and university instructors' well-being during quaranteaching: Findings from a global survey. Frontiers in Psychology, 12, 643229 [Research topic "Covid-19 and Beyond: From (Forced) Remote Teaching and Learning to 'The New Normal' in Higher Education"]. DOI: 10.3389/fpsyg.2021.643229. Supplementary Material: survey.

[illegible]						
I am very punctual.	○	○	○	○	○	○
I finish tasks despite obstacles in the way.	○	○	○	○	○	○
I act according to my conscience.	○	○	○	○	○	○
I am good at helping people work well together.	○	○	○	○	○	○
I find people more stimulating than everything else.	○	○	○	○	○	○
I am a loner.	○	○	○	○	○	○
When something upsets me, I try to keep my emotions in balance.	○	○	○	○	○	○
I use gestures to illustrate what I am trying to say.	○	○	○	○	○	○
When I feel down, I think about what is good in my life.	○	○	○	○	○	○
When I encounter difficult situations, I feel like I am losing control.	○	○	○	○	○	○
I like to learn new skills for my job before they are needed.	○	○	○	○	○	○
When I fail at something that's important to me, I tend to feel alone in my failure	○	○	○	○	○	○
I can manage many things at the same time.	○	○	○	○	○	○
I am willing to take risks.	○	○	○	○	○	○
I get upset easily.	○	○	○	○	○	○
I often lose my temper.	○	○	○	○	○	○
I have difficulty applying my theoretical knowledge in real life situations.	○	○	○	○	○	○
I often make last-minute decisions.	○	○	○	○	○	○
I am always prepared.	○	○	○	○	○	○
I am a perfectionist.	○	○	○	○	○	○
Stress mobilises me.	○	○	○	○	○	○
I like being solely responsible for what I do.	○	○	○	○	○	○
When I'm feeling down, I tend to obsess and fixate on everything that's wrong.	○	○	○	○	○	○

70. **(part 4/4 – almost there!)** *

	completely disagree	mostly disagree	slightly disagree	somewhat agree	mostly agree	completely agree
I take time out for others.	○	○	○	○	○	○
I try to make my group members happy.	○	○	○	○	○	○
I like to spend my free time with others.	○	○	○	○	○	○
I feel embarrassed to have people see my home.	○	○	○	○	○	○
I look directly at my students when talking to them.	○	○	○	○	○	○
Life is beautiful.	○	○	○	○	○	○
I do not think I can control my own future.	○	○	○	○	○	○
I accept challenging tasks.	○	○	○	○	○	○
I can't wait to get started on new projects.	○	○	○	○	○	○
When I feel inadequate in some way, I try to remind myself that feelings of inadequacy are shared by most people.	○	○	○	○	○	○
I have a history of depression.	○	○	○	○	○	○
I can work under pressure.	○	○	○	○	○	○
I always try to make the most of the teaching time.	○	○	○	○	○	○
I continually train to keep my teaching skills and knowledge up-to-date.	○	○	○	○	○	○
I have difficulty keeping my attention on a task.	○	○	○	○	○	○
Every time before I act, I think through the consequences.	○	○	○	○	○	○
I trust my students.	○	○	○	○	○	○
I am a confident user of new technologies.	○	○	○	○	○	○
I am able to find things out by myself.	○	○	○	○	○	○
I believe that good things come to those who wait.	○	○	○	○	○	○
I have no problem having to improvise in class.	○	○	○	○	○	○
I am not interested in other people's problems.	○	○	○	○	○	○
Even the littlest things make me emotional.	○	○	○	○	○	○
People tell me that my emotions are [illegible]	○	○	○	○	○	○

Jelińska, M. and Paradowski, M. B. (2021). The impact of demographics, life and work circumstances on college and university instructors' well-being during quaranteaching: Findings from a global survey. Frontiers in Psychology, 12, 643229 [Research topic "Covid-19 and Beyond: From (Forced) Remote Teaching and Learning to 'The New Normal' in Higher Education"]. DOI: 10.3389/fpsyg.2021.643229. Supplementary Material: survey.

often too intense for the situation.	○	○	○	○	○	○
I am a very sensitive person.	○	○	○	○	○	○
When I am angry/upset, it takes me much longer than most people to calm down.	○	○	○	○	○	○
I get angry at people very easily.	○	○	○	○	○	○

71. How did you find out about this survey? Please try to be specific:

72. If you have any comments about this survey, you may write them here:

73. Thank you very much for filling out this questionnaire! Your responses are very valuable and will help us find out what psychological traits, attitudes and behaviours help educators cope with transitioning to emergency remote instruction. If you would like us to send you the results, you may leave your e-mail address here:

74. Because the epidemic is not yet over, we hope to find out how our participants are doing when the lockdown restrictions have finished and regular classes have resumed. Would you agree to receive a one-time link to a much shorter (12-15-minute-long) mini-questionnaire once schools have returned to face-to-face teaching, or after the end of the semester?

○ Yes – e-mail:

○ No

Thank You!

If you feel that you would like to discuss anything in this survey urgently, you may contact the lead researcher at michal[dot]paradowski[-AT-]uw[dot]edu[dot]pl

Thank you so much once again! If you know friends, colleagues or students who might be willing to fill in the survey, please feel free to forward the relevant links:

survey for EDUCATORS in subjects other than language: xxxxxxxxxxxxxxxxxxxxxxxxxxxxxxxxxxxxxxxxxxxxxxxxxx
survey for LANGUAGE TEACHERS: xxxxxxxxxxxxxxxxxxxxxxxxxxxxxxxxxxxxxxxxxxxxxxxxxxxxxxxx
survey for LANGUAGE LEARNERS: xxxxxxxxxxxxxxxxxxxxxxxxxxxxxxxxxxxxxxxxxxxxxxxxxxxxxxxx
survey for LINGUISTICS INSTRUCTORS: xxxxxxxxxxxxxxxxxxxxxxxxxxxxxxxxxxxxxxxxxxxxxxxxxxxxxxxx
survey for LINGUISTICS STUDENTS: xxxxxxxxxxxxxxxxxxxxxxxxxxxxxxxxxxxxxxxxxxxxxxxxxxxxxxxxx

Stay safe and healthy!

Michał B. Paradowski & Magdalena Jelińska
University of Warsaw
