# Supplementary material for: The Impact of Demographics, Life and Work Circumstances on College and University Instructors’ Well-Being During Quaranteaching
Source: Front Psychol. 2021 Jun 11;12:643229. doi: 10.3389/fpsyg.2021.643229 (PMC8226323; doi:10.3389/fpsyg.2021.643229)
Supplement: Supplementary file 2 [file Data_Sheet_2.docx]

**Appendix: Scales**

**Table 1a. Negative affect scale**

Means, standard deviations, and item-total score-corrected correlations; validity calculated on raw data (*N* = 804, α & ω_h_ = .92, λ_6_ = .90)

| **Item** | **M** | **SD** | **r_tt_** |
| --- | --- | --- | --- |
| I have been feeling irritable. | 3.16 | 1.53 | .77 |
| I feel building up pressure. | 3.26 | 1.61 | .77 |
| I am feeling exhausted all the time. | 3.03 | 1.61 | .75 |
| I have been sad. | 3.35 | 1.62 | .74 |
| I feel tired during the day. | 3.51 | 1.60 | .71 |
| I have been having bouts of anxiety/panic attacks. | 2.62 | 1.64 | .71 |
| I feel I have been losing my sanity. | 2.33 | 1.40 | .68 |
| I feel no motivation to do anything. | 2.63 | 1.53 | .68 |
| I find it harder to get out of bed in the morning. | 2.62 | 1.61 | .64 |
| My dreams have been more emotional/anxious. | 2.87 | 1.60 | .48 |
| My daily life is lacking structure. | 2.77 | 1.50 | .46 |
| **Scale** | **32.17** | **12.71** | **.51** |

*Note:* The introduction to the scale was: “How have you been feeling currently?”

**Table 2a. Situational loneliness scale**

Means, standard deviations, and item-total score-corrected correlations; validity calculated on standardised data (*N* = 804, α = .86, ω_h_ = .85, λ_6_ = .84)

| **Item** | **M** | **SD** | **r_tt_** |
| --- | --- | --- | --- |
| I miss daily conversations with my colleagues. | 2.72 | 2.64 | 2.72 |
| I try to stay in touch with my colleagues every day. | 2.16 | 2.30 | 2.16 |
| I feel lonely. | 1.76 | 2.06 | 1.76 |
| **Scale** | **6.64** | **3.18** | **.68** |

*Note:* The introduction to the scale was: “To what extent do you agree with the following statements:”

**Table 3a. Situational anxiety scale**

Means, standard deviations, and item-total score-corrected correlations; validity calculated on raw data (*N* = 804, α = .81, ω_h_ = .82, λ_6_ = .80).

| **Item** | **M** | **SD** | **r_tt_** |
| --- | --- | --- | --- |
| I feel anxious about my future. | 3.62 | 1.59 | .72 |
| I worry about my job stability. | 3.39 | 1.73 | .67 |
| I worry about my family/friends. | 4.43 | 1.39 | .55 |
| I worry about housing instability. | 2.49 | 1.54 | .54 |
| I am afraid of an approaching economic crisis. | 4.80 | 1.22 | .54 |
| The current crisis will further social inequalities. | 5.01 | 1.17 | .37 |
| **Scale** | **23.74** | **6.2** | **.41** |

*Note:* The introduction to the scale was: “To what extent do you agree with the following statements:”

**Table 4a. Family and social support scale**

Means, standard deviations, and item-total score-corrected correlations; validity calculated on raw data (*N* = 804, α & ω_h_ = .80, λ_6_ = .78).

| **Item** | **M** | **SD** | **r_tt_** |
| --- | --- | --- | --- |
| I feel comfortable having my family/partner/roommates/flatmates around during this time. | 4.34 | 2.14 | .76 |
| I have good relations at home. | 4.63 | 2.12 | .72 |
| I enjoy being able to spend more time with my family/children/partner. | 3.95 | 2.27 | .65 |
| I feel the support of my family/flatmates/friends/community during this time. | 4.68 | 1.67 | .38 |
| **Scale** | **17.61** | **6.56** | **.52** |

*Note:* The introduction to the scale was: “To what extent do you agree with the following statements:”
